# Walking Among Pioneers – Sperm DNA Fragmentation and a Growing Focus on Male Factor Infertility

**DOI:** 10.1590/S1677-5538.IBJU.2024.9925

**Published:** 2025-01-10

**Authors:** Robert Matthew Coward

**Affiliations:** 1 Atlantic Reproductive Medicine Raleigh North Carolina United States Atlantic Reproductive Medicine, Raleigh, North Carolina, United States

## COMMENT

Ask a medical student to name some pioneers in medicine, and you may hear about Louis Pasteur, Marie Curie, Alexander Fleming, or Jonas Salk. Ask a surgeon, and you'll hear about Joseph Lister, Harvey Cushing, or Michael DeBakey. Most urologists will also mention heroes from the early 20th century such as Hugh Hampton Young, Frederic Foley, or Charles Huggins and the Nobel Prize. Most of the greatest innovations in medicine and urology were before the turn of the last century.

The field of reproductive urology and male infertility, on the other hand, is still in the dawn of its era. The first child conceived using in vitro fertilization (IVF) technology was born in 1978, and Gianpiero Palermo first described intracytoplasmic sperm injection (ICSI) in 1992. IVF with ICSI was the catalyst that provided the first real opportunity for men with severe male factor infertility to become biological fathers. Shortly thereafter, Peter Schlegel described the microdissection testicular sperm extraction (micro-TESE) in 1999, representing the single greatest advancement in the surgical treatment of severe male factor infertility of our lifetimes. Many of the pioneers of our subspecialty are still currently in practice in 2024.

The Society for Male Reproduction and Urology (SMRU) was founded in 1995 by Marc Goldstein and Dorrie Lamb with the mission "to promote the advancement of our understanding of male reproductive physiology and management of male infertility by providing a forum for the dissemination of both basic and clinical research information and support of educational programs." As a Society, one of the highest honors we have is the coveted invitation for the American Urological Association (AUA)'s Bruce Stewart Lecture, given annually at the American Society for Reproductive Medicine (ASRM) Congress. The 2024 AUA Bruce Stewart Lecture was given at the ASRM Congress on October 23, 2024, in Denver, Colorado, USA, by Dr. Sandro Esteves, titled "From Double Helix to Double Trouble: Sperm DNA Fragmentation Unveiled" (1).

Dr. Esteves is a true pioneer in the field. He is a founding member of the SMRU, and he is the founder of the world's first (and still only) comprehensive center of reproductive medicine with a primary focus on caring for couples with male factor infertility. With over 350 peer-reviewed publications, his contributions to the field of reproductive urology through innovative research are immeasurable. His most substantial work has come in the area of sperm DNA fragmentation and its influence on a couple's infertility. In fact, no other researcher across the globe has made a greater impact on this critically important topic than Dr. Esteves.

In the accompanying manuscript, reflecting Dr. Esteves's lecture mentioned above, sperm DNA fragmentation and its timely relevance for the field of reproductive medicine is described in perfect detail. He lends his research group's expertise in a friendly, helpful voice that only he can do. He makes the concepts crystal clear for the reader, even providing enough detail for implementation into one's clinical practice if they have yet to do so. Although the recognition of sperm DNA integrity is one of the most important innovations in the field of male reproductive urology in the 21st century, sperm DNA fragmentation testing is only one tool in a growing armamentarium of diagnostics we now have to characterize male factor infertility beyond the semen analysis. Dr. Esteves concludes in this manuscript that, ultimately, finding a balance between the attention provided to female and male factor infertility is the best path to the most optimal outcomes for the couples we serve.

I encourage all of my reproductive urology colleagues worldwide to join the ASRM and the SMRU (https://connect.asrm.org/smru/aboutus/membershipbenefits) so that they can access the latest research, education, and opportunities for growth as reproductive medicine providers. As President of the SMRU from 2024-2025, I extend heartfelt congratulations to Dr. Sandro Esteves, the 2024 AUA Bruce Stewart Lecturer for the ASRM Congress. The field of male reproductive medicine is young, allowing the next generation of leaders to always be walking among pioneers like Dr. Esteves.

**Figure:**
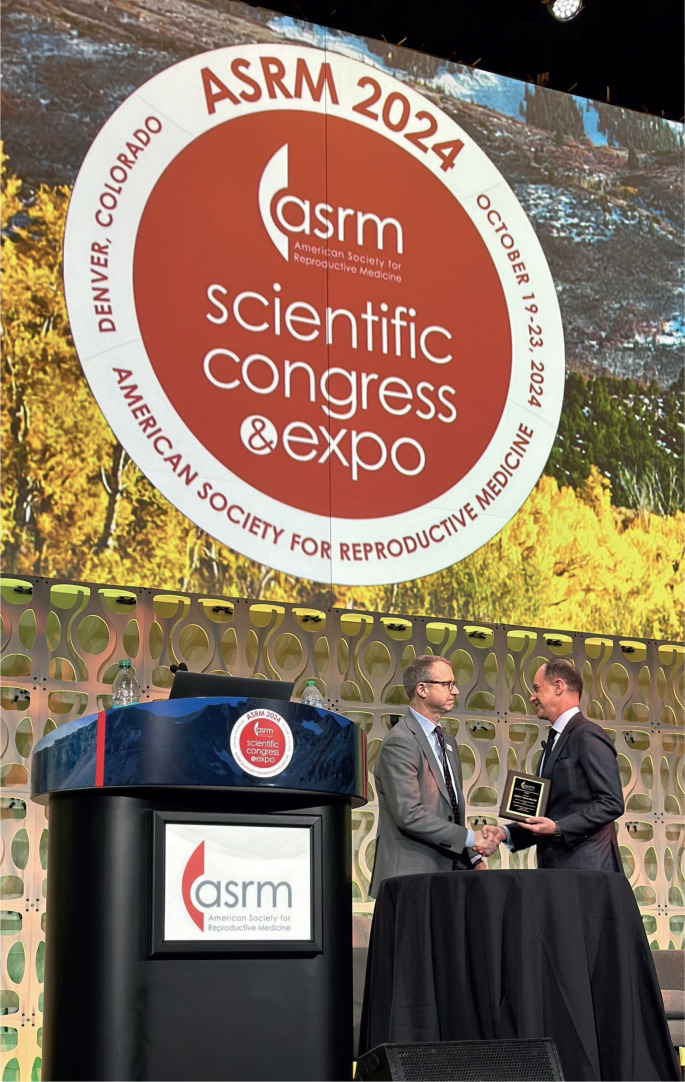
Photograph of Dr. Sandro Esteves receiving a recognition plaque from Dr. Robert Brannigan, honoring his exceptional contributions as the 2024 ASRM Reproductive Urology Keynote Lecturer.
